# Microbiome as Mediator of Diet on Colorectal Cancer Risk: The Role of Vitamin D, Markers of Inflammation and Adipokines

**DOI:** 10.3390/nu13020363

**Published:** 2021-01-25

**Authors:** Davide Serrano, Chiara Pozzi, Silvia Guglietta, Bruno Fosso, Mariano Suppa, Patrizia Gnagnarella, Federica Corso, Federica Bellerba, Debora Macis, Valentina Aristarco, Paolo Manghi, Nicola Segata, Cristina Trovato, Maria Giulia Zampino, Marinella Marzano, Bernardo Bonanni, Maria Rescigno, Sara Gandini

**Affiliations:** 1 Division of Cancer Prevention and Genetics, IEO European Institute of Oncology IRCCS, 20125 Milan, Italy; debora.macis@ieo.it (D.M.); valentina.aristarco@ieo.it (V.A.); bernardo.bonanni@ieo.it (B.B.); 2 Department of Experimental Oncology, IEO European Institute of Oncology IRCCS, 20125 Milan, Italy; chiara.pozzi@humanitasresearch.it (C.P.); gugliett@musc.edu (S.G.); Federica.Corso@ieo.it (F.C.); Federica.Bellerba@ieo.it (F.B.); maria.rescigno@hunimed.eu (M.R.); sara.gandini@ieo.it (S.G.); 3 Humanitas Clinical and Research Center–IRCCS, Mucosal Immunology and Microbiota Unit, ViaManzoni 56, Rozzano, 20125 Milan, Italy; 4 Department of Microbiology and Immunology, Medical University of South Carolina and Hollings Cancer Center, Charleston, SC 29425, USA; 5 Institute of Biomembranes, Bioenergetics and Molecular Biotechnologies, Consiglio Nazionale delle Ricerche, 70128 Bari, Italy; b.fosso@ibiom.cnr.it (B.F.); m.marzano@ibiom.cnr.it (M.M.); 6 Department of Dermatology, Université Libre de Bruxelles, Hôpital Erasme, 1060 Brussels, Belgium; dr.marianosuppa@gmail.com; 7 Department of Medicine, Institut Jules Bordet, 1000 Brussels, Belgium; 8 Division of Epidemiology and Biostatistics, IEO European Institute of Oncology IRCCS, 20125 Milan, Italy; patrizia.gnagnarella@ieo.it; 9 Department CIBIO, University of Trento, 38122 Trento, Italy; paolomanghi1974@gmail.com (P.M.); nicola.segata@unitn.it (N.S.); 10 Division of Endoscopy, IEO European Institute of Oncology IRCCS, 20125 Milan, Italy; cristina.trovato@ieo.it; 11 Division of Medical Oncology Gastrointestinal and Neuroendocrine Tumors, IEO European Institute of Oncology IRCCS, 20125 Milan, Italy; maria.zampino@ieo.it

**Keywords:** microbiota, diet, colorectal cancer, obesity, inflammation, vitamin D

## Abstract

Obesity and diet are associated with colorectal cancer (CRC) risk, and microbiome could mediate this risk factor. To investigate this interaction, we performed a case–control study (34 CRC cases and 32 controls) and analyzed fecal microbiota composition using 16S rRNA metabarcoding and sub-sequential shotgun analyses of genomic bacterial DNA to evaluate the role of microbiome and diet in CRC etiology, taking into account vitamin D and other risk biomarkers. Dietary habits were evaluated using a short questionnaire. Multivariate methods for data integration and mediation analysis models were used to investigate causal relationships. CRC cases were significantly more often deficient in vitamin D than controls (*p* = 0.04); *FokI* and *CYP24A1* polymorphism frequency were different between cases and controls (*p* = 0.03 and *p* = 0.02, respectively). A diet poor in fatty fish and rich in carbohydrates was found to be significantly associated with CRC risk (*p* = 0.011). The mediation analysis confirmed the significant role of the microbiome in mediating CRC risk—increasing levels of *Bifidobacteria*/*Escherichia* genera ratio, an indicator of “healthy” intestinal microbiome, can overcome the effect of diet on CRC risk (*p* = 0.03). This study suggests that microbiome mediates the diet effect on CRC risk, and that vitamin D, markers of inflammation, and adipokines are other factors to consider in order to achieve a better knowledge of the whole carcinogenic process.

## 1. Introduction

Colorectal cancer (CRC) is the third most commonly diagnosed cancer type in humans [1]. Epidemiological studies have shown that CRC patients exhibit common risk factors such as obesity, low physical activity, smoking, high alcohol consumption, high caloric intake, and a diet extremely rich in red meat and low in vegetables [2,3].

In this context, we have previously found a link between carbohydrate-rich diets (with a high glycemic index and glycemic load) and CRC in humans [4]. The gut microbiota represents another important determinant for CRC development, progression, and response to therapy [5] and recently two pooled analyses [6,7] identified reproducible microbiome biomarkers and accurate disease-predictive models, which can set the basis for screening tests and hypothesis-driven mechanistic and prognostic studies. Furthermore, recent reports showed that in DNA mismatch repair-deficient mice, a high carbohydrate diet, metabolized by a microbiota particularly enriched in *Firmicutes families*, drives aberrant proliferation and accelerated polyp formation [8]. This provides evidence that interaction between diet and microbiota can affect CRC development and progression.

Among known the risk factors for CRC, there are high levels of inflammatory markers such as C-reactive protein [9,10], or cytokines, such as interleukin (IL)-6, which may promote tumor initiation and progression [11]. Notably, adipose tissue can regulate inflammation and insulin sensitivity via the secretion of several adipokines such as adiponectin [12]. Reduction of adiponectin occurs in obese patients and its levels are indirectly correlated with cancer [13]. Besides lowering adiponectin and fueling inflammation, obesity can result in vitamin D sequestration, therefore contributing to low vitamin D levels, which has been associated with many chronic diseases including cancer [14]. Several lines of evidence suggest that vitamin D has an important role in regulating inflammation [15]. Dietary vitamin D supplementation significantly lowered inflammatory cytokines in mouse models of bacteria-driven colon cancer [16] and has been linked to decreased circulating proinflammatory cytokines in patients with colorectal adenomas [17]. Furthermore, we observed a significant reduction in CRC risk comparing the highest versus the lowest level of serum 25-hydroxycholecalciferol (25-OHD or 25-hydroxy-vitamin D), with a significant dose–response effect [18] and a significant association with vitamin D receptor polymorphisms [19]. A recent meta-analysis showed that vitamin D supplementation significantly reduced total cancer mortality [20]. Furthermore, vitamin D status can influence the intestinal microbiota by promoting anti-inflammatory responses and inhibiting infections [21,22], and vitamin D supplementation changes the microbiota of the upper gastrointestinal tract [23]. This suggests that vitamin D signaling may inhibit CRC by altering the colonic microbiota and reducing secondary bile acid levels.

Despite this evidence, to date, there is a lack of clinical studies simultaneously evaluating the interplay between multiple risk factors and CRC. In this study, we aimed at evaluating the role of microbiome and diet in CRC etiology, taking into account lifestyle and other risk biomarkers such as vitamin D levels, dietary intake, body mass index (BMI), inflammatory markers, and adipokines. Importantly, we carried out a multivariable and multivariate analysis for data integration in a prospective study and we employed mediation modeling, which is a critical tool used in molecular epidemiology to infer causal pathways for biological processes.

## 2. Materials and Methods

### 2.1. Participants and Study Design

A total of 84 subjects were recruited and screened at the European Institute of Oncology (Milan, Italy), including 34 CRC cases and 32 controls. Cases (recent CRC diagnosis) were aged between 35 and 70 years and were recruited before surgery or neoadjuvant treatment for resectable CRC. Main exclusion criteria were previous history of any cancer (5 years, other than cervical intraepithelial neoplasia or non-melanoma skin cancer), presence of mutations known to be associated to familial CRC (familial adenomatous polyposis, Lynch syndrome), current daily supplementation of vitamin D or calcitriol or high dose of calcium, history of malabsorption syndrome or any chronic inflammatory bowel disease (IBD), use of antibiotics in the last 6 weeks, chronic alcoholism, and any medical condition that in the physician’s opinion could potentially interfere with vitamin D metabolism. Controls were subjects who underwent a recent negative colonoscopy, with no other relevant gastrointestinal disorders. Initially we conducted our enrollment trying to match for age (±5 years) and season at blood collection (±2 months). However, since we lost 2 patients, the final study cannot be considered any longer a matched case–control study. Additionally, healthy subjects with a family history of CRC are over-represented, especially among younger subjects, as they undergo colonoscopy more frequently for screening purposes.

The study (IEO #118) was approved by the Institutional Review Board (European Institute of Oncology Ethical Committee), and all subjects gave their written informed consent according to ICH-Good Clinical Practice.

### 2.2. Circulating Biomarkers

Morning fasting blood samples were collected at baseline. Serum was separated by 10 min of centrifugation at 1350× *g* and stored at −80 °C for subsequent biomarker quantification. Serum concentrations of 25-hydroxy-vitamin D (25-OHD) were measured by a chemiluminescence microparticle immunoassay (CMIA) designed for the automated instrument Architect (Abbott Diagnostics, Lake Forest, IL, USA). Due to high seasonal variability, different cut-off points were considered to define 25-OHD deficiency in different seasons (<20 ng/mL in summer/autumn and <10 ng/mL in winter/spring). For the high-sensitivity C-reactive protein (hs-CRP) analysis, we employed a latex immunoturbidimetric high-sensitivity method on the same instrument. IGF-II was measured by sandwich ELISA from Mediagnost (Bensheim, Germany). IGFBP-3, IL-6, vitamin D binding protein (VDBP), leptin, and adiponectin were determined by ELISA (R&D Systems). Serum zonulin was determined using an ELISA kit from Elabscience (Wuhan, China). Many samples, including all control subjects, had IL-6 levels below the lowest standard (3.13 pg/mL). We assigned the lowest detectable value of 3.13 pg/mL to these samples to run the statistical analysis.

### 2.3. Single Nucleotide Polymorphism (SNPs) Analysis

Genomic DNA was extracted from whole blood specimens using a QIAamp DNA blood kit (Qiagen, Valencia, CA, USA), according to the manufacturer’s instructions on the automated platform “QIAcube” (Qiagen, Valencia, CA, USA), and quantified using NanoDrop spectrophotometer (Thermo Scientific, Wilmington, DE, USA). DNA samples were genotyped for a comprehensive set of single nucleotide polymorphisms (SNPs). We analyzed *Bsm1* (rs1544410), *Taq1* (rs731236), *Fok1* (rs228570), and *Apa1* (rs7975232) in the VDR gene; 3 SNPs involved in vitamin D metabolism (CYP24A1-rs6013897, CYP27B1-rs10877012, CYP2R1-rs10741657); and rs2282679, rs7041, and rs4588 in the GC gene coding for the main transporter of vitamin D in the circulation. SNPs genotyping was performed by the TaqMan SNP Genotyping Assays using an ABI PRISM 7500 FAST Real-Time PCR System (Thermo Fisher Scientific). Briefly, nearly 10 ng of DNA in 2 μL was added to a 10-μL reaction well together with 8 μL of reaction mix containing forward and reverse primers and 2 allele-specific fluorescent labelled probes (1 wild-type and 1 variant allele-specific). Control samples, representing a complete set of genotypes for all SNPs, were processed in each run. Hardy–Weinberg equilibrium (HW) for genotype frequencies was tested using a chi-squared test in controls.

### 2.4. Microbiota Analysis

Freshly voided stool samples were collected from controls and cases (before surgery, or any other treatment). Stool samples were transported refrigerated to the laboratory within 6 hours from collection and immediately frozen at −80 °C.

For metagenomic analysis, genomic bacterial DNA was isolated from feces of CRC patients and healthy donors with the G’NOME isolation kit (MP Biomedicals) following a published protocol [24]. The V5-V6 hypervariable regions of 16S rRNA gene were amplified and sequenced using the Illumina MiSeq platform, following library preparation and sequencing procedures previously described [25]. Principal Component Analysis PCA analysis highlighted how data variability is not related the sequencing but mainly due to inter-subject variability. Moreover, the taxonomic classification at phylum level shows a higher agreement in intraclass correlation coefficient test (0.98; 95% CI: 0.95–0.99) between the 2 sequencing runs, highlighting again the absence of any batch effect. Whole metagenome shotgun sequencing [26] was applied on the same DNA samples used for 16S rRNA gene sequencing. Metagenomic libraries were generated with a Nextera XT DNA Sample Prep Kit (Illumina, San Diego, CA, USA) and sequencing was carried out on the HiSeq2500 platform (Illumina) at a targeted depth of 5.0 Gb (100-bp paired end reads). Shotgun metagenomics sequencing samples were pre-processed as previously described [6].

### 2.5. Dietary Assessment

The subjects’ habitual diet before the enrollment was assessed using a short questionnaire adapted from a new validated questionnaire [27]. This questionnaire evaluates the consumption of food groups commonly present in the diet of the Italian population such as milk and yogurt; bread, pasta, and cereals; meat and meat products; cheese; fish; eggs; vegetables; fruit; and sweets, pastries, and biscuits. Subjects were asked to indicate the typical average weekly frequency of consumption for each food group. The questionnaire was tested to measure the adherence of a Mediterranean pattern, and the correlation coefficients between the consumption frequencies of the short questionnaire and the daily consumption of the corresponding food items assessed from the European Prospective Investigation into Cancer and Nutrition food frequency questionnaire (FFQ) [28] ranged from poor to very high. Correlation coefficients were found from moderate to very high for fats, vegetables, cereals, white meat, sweet and cakes, red meat, fresh fruits, fish, dried fruits, pulses, soft drinks, milk and yoghurt, and wine consumption. This questionnaire was adapted to investigate vitamin D consumption. The adaptation consisted of adding 1 question to discriminate between consumption of fatty fish and other types of fish so as to evaluate the consumption of vitamin D. The present study represents a first validation investigating the association of 25-OHD with fatty fish consumption. The questionnaire includes 5 consumption levels ranging from “never or seldom” to “high frequency”, depending on the type of food groups (daily or weekly consumption). For each question, a standard portion is also indicated to help reporting consumption as accurately as possible. To avoid sparse data, we reported and analyzed food intake by grouping the answers according to the sample size in the various categories. Categories of different food groups were grouped in order to identify high-risk consumption: for sweets, we compared “twice/week” vs. “lower consumption”, as suggested by a previous study [29]; for meat, we compared “at least twice/week” vs. “lower consumption” following the World Cancer Research Fund International (WCRF) [30]; for dairy products, we compared “once/day” vs. “lower consumption” following WCRF recommendations [31].

### 2.6. Statistical Analysis

Due to the length of the manuscript, we provide all related information in the Supplementary Materials available on the journal website.

## 3. Results

### 3.1. Risk Factors and Serum Biomarkers

Demographic, epidemiological, and clinical characteristics at baseline for cases (CRC patients) and controls (healthy individuals) are summarized in Table 1. Expectedly, several risk factors were associated with CRC. Compared to controls, we found significantly more cases amongst those with BMI > 25 (66.7% vs. 37.5% for cases and controls, respectively; *p*-value = 0.02), performing less physical activity (42.2% vs. 75.0% for cases and controls, respectively; *p*-value = 0.006), consuming alcohol regularly (85.3% vs. 53.1% for cases and controls, respectively; *p*-value = 0.005), and who are smokers (64.8% vs. 25.0% for cases and controls, respectively; *p*-value = 0.005). We did not find any significant difference between cases and controls in terms of comorbidities (such as diabetes and hypercholesterolemia) and recent use of drugs (such as metformin, aspirin, and statin; data not shown). Several serum biomarkers were significantly different in cases and controls (Table 2). Cases had higher hs-CRP (>0.1; 79.4% vs. 50.0% for cases and controls, respectively; *p*-value = 0.012), lower adiponectin (≤6; 58.8% vs. 21.90% for cases and controls, respectively; *p*-value = 0.002), and higher IL-6 (>4; 26.5% vs. 6.3% for cases and controls, respectively; *p*-value = 0.03). We also confirmed that cases were more often significantly deficient in vitamin D, relative to the season, than controls (29.4% vs. 9.4% for cases and controls, respectively; *p*-value = 0.04; Table 2). As shown in Supplementary Table 1, where we report 25-OHD levels by season and CRC status, in springtime (March to June) levels of 25-OHD were very low (<20 ng/mL) for both cases and controls and lower in cases than controls. Similar differences between cases and controls were found all year long and also throughout summer–autumn (July to October) when levels of 25-OHD were >20 ng/mL.

### 3.2. Microbiome Biomarkers and Functional Profiles

**Figure 1:**
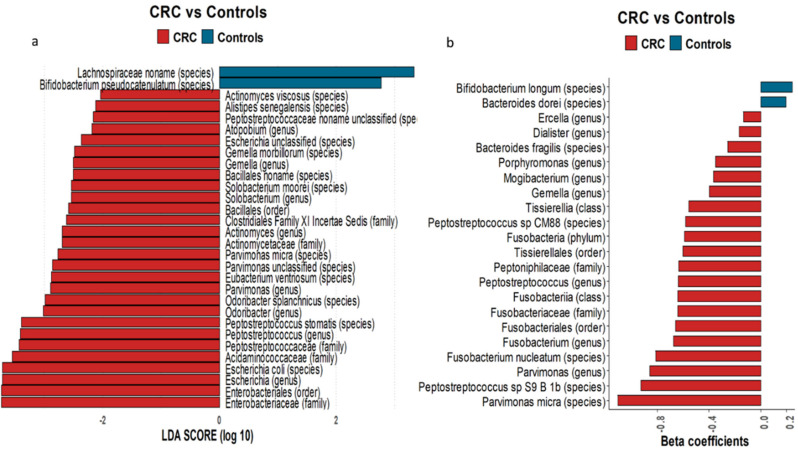
Microbiome composition in colorectal cancer patients and healthy controls. (**a**) Bar plot representing the result obtained by applying linear discriminant analysis effect size (LEfSe) on metabarcoding shotgun data. The bar length represents the linear discriminant analysis (LDA) score as a measure of the significant differences between the CRC (red) and control (blue) subjects (LDA score > 2). (**b**) Bar plot representing the taxa associated with CRC obtained through applying multivariable logistic model on metabarcoding 16S data, adjusted for age, smoking, and alcohol consumption. The bar length represents the significant beta-coefficient as a measure of the association with CRC (red) or healthy control (blue) subjects (*p* < 0.05).

Since microbiota are an important determinant of CRC development and progression, we performed shotgun metagenomic analysis to characterize the fecal microbiota in cases and controls (Figure 1a). In line with previously published reports, our data showed a significantly higher abundance of *Escherichia coli*, *Parvimonas micra*, and *Solobacterium moorei* species (an oral bacterium typical of periodontal disease) in cases [32]. Conversely, operational taxonomic units (OTUs) corresponding to the butyrate-producing *Lachnospiraceae* family and probiotic species, such as *Bifidobacterium longum*, were significantly enriched in controls. After adjusting for confounders such as age, smoking, and alcohol consumption using multivariate logistic models on metabarcoding 16S data, we found that the profile of gut microbiota reported among controls was consistent with the general profile of the human gut microbiota, dominated by *Bacteroidetes* and *Bifidobacterium* species, which have health-promoting properties [6,7] (Figure 1b). In CRC patients, we found significant associations with *Parvimonas micra*, *Fusobacterium nucleatum*, and *Bacteroides fragilis* species. In addition, significantly higher expression of metabolic pathways associated with gluconeogenesis, putrefaction, and fermentation was detected in cases (data not shown).

### 3.3. Interplay between Vitamin D, Dietary Habits, and Microbiota in CRC

We investigated the association of CRC with vitamin D by analyzing the following markers: 25-OHD, serum VDBP, VDR, and *GC* (that encoded VDBP) polymorphisms and polymorphisms of vitamin D-metabolizing enzymes (*CYP24A1*, *CYP27B1*, and *CYP2R1*), and consumption of cholecalciferol-rich fatty fish. We then analyzed the interaction between each of these markers with microbiota and CRC status.

As shown in Table 3, we found significantly more subjects with *ff FokI* polymorphism among cases (20.6% vs. 3.1% for cases and controls, respectively, *p*-value = 0.03) and more subjects with *AA CYP24A1* polymorphism among cases (14.7% vs. 0% for cases and controls, respectively, *p*-value = 0.02).

We also found a significantly greater percentage of CRC patients with high (<2) abundance of *Parvimonas genus*, particularly in cases with low levels of 25-OHD (insufficient relative to the season) (*p* = 0.0002 Wilcoxon rank test; Supplementary Figure S1).

In order to assess the correlation between risk factors such as obesity, diet, lifestyle, microbiota, and CRC, we asked cases and controls to report their dietary habits by filling in a short validated dietary questionnaire [27]. A dose–response trend of 25-OHD with increasing consumption of fatty fish among control subjects was found (Supplementary Table S3). Cases displayed a significantly greater consumption of pasta, rice, and bread (food rich in carbohydrates) (67.6% vs. 28.1% for cases and controls, respectively; *p*-value = 0.001) and a significantly reduced consumption of fatty fish (salmon, herring, mackerel) (11.8% vs. 34.4% for cases and controls, respectively, for 2–3 times a week; *p*-value = 0.03; Supplementary Table S4).

The odds ratio by lifestyle variables and dietary scores analyzed as continuous variable obtained from the multivariable logistic model as well as categorical (low fatty fish and high carbohydrate/cereals intake) are shown in Table 4. This model (low in fatty fish and high in carbohydrate/cereals) reached a significant odds ratio (OR) of 5.88 (95% CI: 1.49–25.0; *p* = 0.011) adjusting for confounders (Table 4). Moreover, through applying WCRF guidelines (high physical activity and a healthy diet—high consumption of fruit and vegetables, or low consumption of meat or sweets, cakes, and pastries), we found a significant inverse association with CRC. Subjects following these guidelines reached 87% decreased risk of CRC (OR = 0.23 (95% CI: 0.08–0.67; *p* = 0.007)), adjusting for confounders (Table 4).

**Figure 2:**
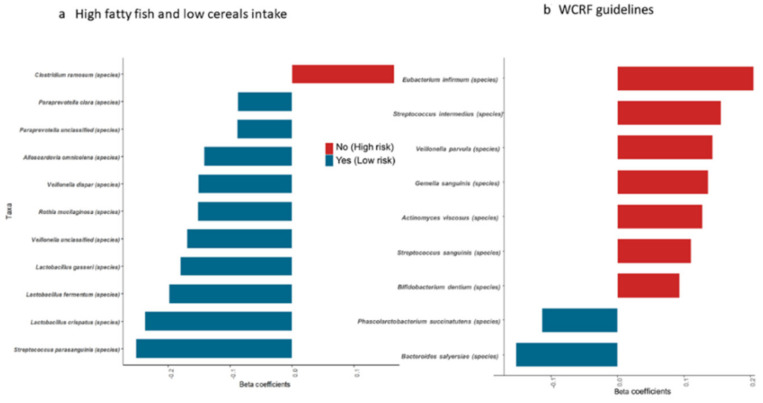
Species associated with diet and World Cancer Research Fund International (WCRF) guidelines. Results from logistic models of shotgun data. Bar plot representing the result obtained by applying multivariable logistic models, adjusted CRC status, age, and sex. The bar length represents the significant beta-coefficients of the models (*p* < 0.05). High-risk diet or not following the WCRF (red) and low-risk diet or following WCRF (blue). “Yes” indicates low-risk diet—“high fatty fish and low carbohydrates/cereals”; “No” indicates high-risk diet. “Yes” indicates those who follow WCRF guidelines; “No” indicates those who do not follow WCRF guidelines. (**a**) for high fatty fish and low cereals intake. (**b**) for adherence to WCRF guideline.

Subsequently, we investigated whether specific bacterial taxa were enriched in subjects adhering to WCFR recommendation or to other dietary habits. We carried out the analysis on the basis of shotgun data with multivariable logistic models, adjusted for CRC status, age, and sex. As shown in Figure 2a, a diet including “high fatty fish and low cereals/carbohydrate intake” was more significantly associated with *Lactobacillus* species. An opposite diet (low in fatty fish and high in cereals/carbohydrates) showed stronger association with *Clostridium ramosum* (belonging to the Firmicutes phylum). Subjects who did not follow WCRF guidelines showed an enrichment in species belonging to the oral microbiome such as *Streptococcus sanguinis* (Figure 2b).

### 3.4. Microbiome-Mediated Diet Effect on CRC Risk

**Figure 3:**
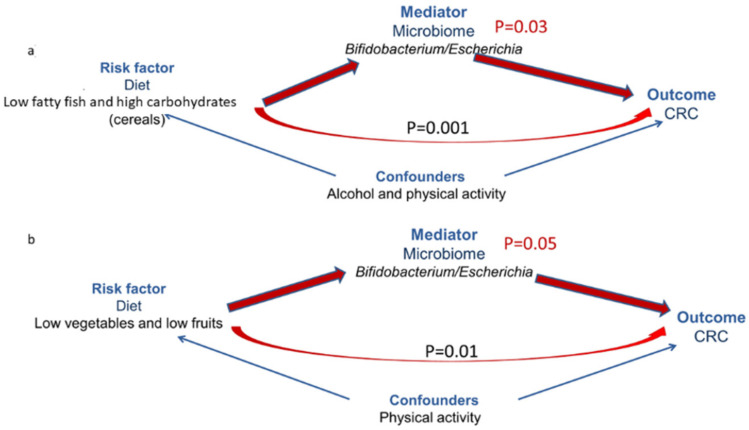
Direct acyclic graph of mediation model analyses. Microbiome as mediator of the effect of “low fatty fish and high cereals/carbohydrates” for CRC risk. In red, natural indirect effect (NIE) and natural direct effect (NDE); in blue, the effect of confounders on exposure–outcome relationship. *p*-value obtained from mediation analysis. Direct effect of diet: highly significant positive association with CRC risk (odds ratio (OR) = 17 (95% CI: 3.4–91); *p* = 0.001). Microbiome (*Bifidobacterium/Escherechia* genera) significantly mediates the effect of diet (OR = 0.31 (95% CI: 0.10–0.94); *p* = 0.03), decreasing the risk with increasing value of *Bifidobacterium/Escherichia*. NIE = natural indirect effect; NDE = natural direct effect. (**a**) for low fatty fish and low cereals intake. (**b**) for low vegetable and low fruits intake.

To understand whether diet-induced differences in microbiome correlate with CRC, we conducted a mediation analysis. We assessed both the direct causal effect of the diet on CRC outcome and the indirect causal effect (through the microbiome) using an acyclic graph, which analyzes whether high-risk diet effect is significantly mediated by microbiome composition or is independent. We assumed no interaction between high-risk diet and microbiome as it was not statistically significant in multivariate analysis. As shown in Figure 3, we found that in subjects consuming a “low fatty fish and high carbohydrates/cereals” diet (associated with a higher CRC risk), there was a significant 70% reduction of CRC risk at increasing levels of the log-transformed ratio of *Bifidobacteria* over *Escherichia* genera (*p* = 0.03)—the OR of the indirect effect through microbiome was 0.31 (95% CI: 0.10–0.94), adjusting for significant confounders (alcohol intake and physical activity). The direct effect was also significant (*p* = 0.001), as well as the total effect of the diet on CRC (*p* = 0.03).

We also carried out a similar analysis considering BMI as a mediator and found that the indirect effect through the microbiome was not statistically significant (*p* = 0.73). The only significant effect was the direct effect of diet (*p* = 0.002), suggesting that diet and obesity are independent risk factors for CRC. We found similar results when considering low levels of adiponectin as mediator (*p* = 0.30). Moreover, in this case, the only significant effect was the direct effect of diet (*p* = 0.005). We also evaluated the ratio between the genera *Firmicutes* and *Bacteroides*, but the indirect effect considering this ratio was not significant (OR = 0.96 (95% CI: 0.13–6.80; *p* = 0.97), and the only significant effect was the direct effect of diet (*p* = 0.003) (data not shown).

Overall, these results indicate that although the diet may clearly influence microbiome and the risk of CRC, the composition of the microbiome may still protect the individual from CRC development independently on the type of diet. Therefore, the microbiome should be taken into account in preventive or therapeutic strategies.

### 3.5. Integrative Data Analysis

**Figure 4:**
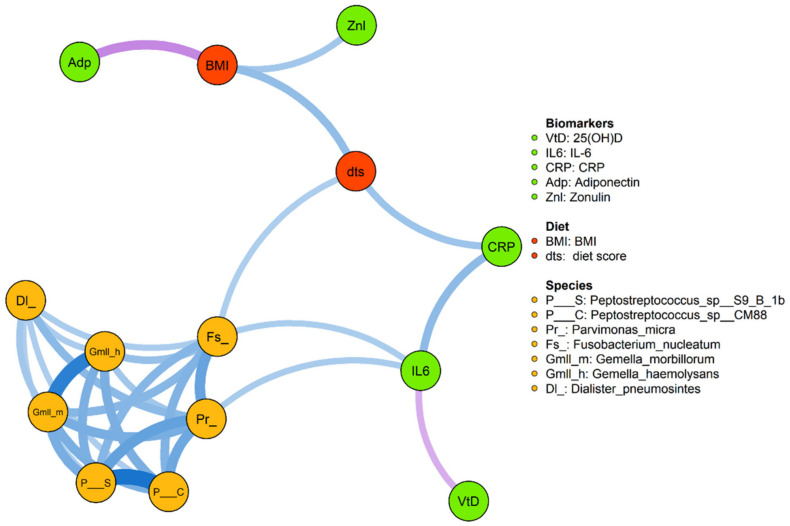
Correlation network analysis among serum markers, BMI, dietary score, and CRC-associated species. The width of each edge corresponds to the absolute values of Spearman correlation coefficients and the transparency of edge represents an adjusted *p*-value. The line color indicates the direction of a correlation (blue for positive and violet for negative). The relative size of the node was determined by the relative abundance of the microbe. Correlations with *p*-values less than 0.05 are displayed. Relevant Spearman correlation coefficients: BMI and diet score (*R* = 0.41, *p* < 0.001), diet score and high-sensitivity C-reactive protein (hs-CRP) (*R* = 0.37, *p* = 0.002), 25-hydroxycholecalciferol (25-OHD) and hs-CRP (*R* = −0.34, *p* = 0.005), 25-OHD and interleukin (IL)-6 (*R* = 0.27, *p* = 0.0027), *Fusobacterium nucleatum* and IL-6 (*R* = 0.31, *p* = 0.01), adiponectin and BMI (*R* = −0.51, *p* < 0.001), BMI and zonulin (*R* = 0.36, *p* = 0.004). CRP = hs-CRP.

To investigate the correlation between BMI, serum inflammatory biomarkers (IL-6 and hs-CRP), 25-OHD, adiponectin, and the continuous dietary score and CRC-associated taxa, we first conducted a network analysis based on statistically significant correlations (Figure 4, all *p* < 0.05). Our results showed that there was a positive correlation between the BMI and the dietary score (*R* = 0.41 and *p* < 0.001), and the dietary score positively correlated with hs-CRP (*p* = 0.37 *p* = 0.002) and IL-6 (*R* = 0.27 and *p* = 0.027). Conversely, inflammatory markers inversely correlated with 25-OHD (with hs-CRP: *R* = −0.34 and *p* = 0.005) and positively correlated with *F. nucleatum* (with IL-6: *R* = 0.31 and *p* = 0.01) and other bacterial species associated with CRC. Adiponectin showed a significant inverse correlation with BMI (*R* = −051 and *p* < 0.001), while BMI positively correlated with zonulin (*R* = 0.36 and *p* = 0.004), a protein that modulates intestinal barrier function.

**Figure 5:**
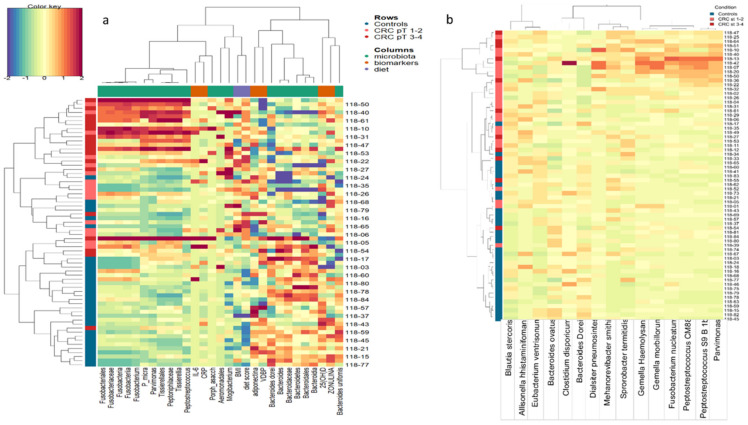
Data integration. (**a**) Heatmap for data integration. Plot generated by performing a sparse partial least square-differential analysis (sPLS-DA) (10-fold cross-validation and 100 repeats) and selecting the most discriminative species, serum biomarkers, BMI, and diet score. (**b**) Heatmap plot generated by performing a sparse partial least squares differential analysis (sPLS-DA) (10-fold cross-validation and 100 repeats) and selecting the most discriminative species by using the first and second component loading vectors.

In order to investigate how microbiota taxa interact with serum inflammatory biomarkers, vitamin D status indicators such as 25-OHD and VDBP, adiponectin, and zonulin, we used canonical correspondence analysis (CCA). In the triplot (Supplementary Figure S2), each factor’s weight is proportional to its arrow length. The first component of the CCA was the only one that was statistically significant (*p* = 0.001) and correlated negatively with IL-6 and hs-CRP in CRC cases, and positively with 25-OHD, VDBP, and adiponectin in healthy subjects. We also observed that the effect of zonulin was weaker compared to other serum biomarkers, as indicated by the lengths of the vectors. *F. nucleatum*, *Parvimonas micra*, and *Porphyromonas* positively correlated with hs-CRP and IL-6, whereas *Bacteroides dorei* and *Bifidobacterium longum* positively correlated with 25-OHD and adiponectin. Data Integration Analysis for Biomarker Discovery (DIABLO) including taxa, serum biomarkers, BMI and dietary factors, allowed us to better discriminate between CRC patients and healthy controls (Figure 5a), compared to where we included only gut microbiome taxa (Figure 5b).

**Figure 6:**
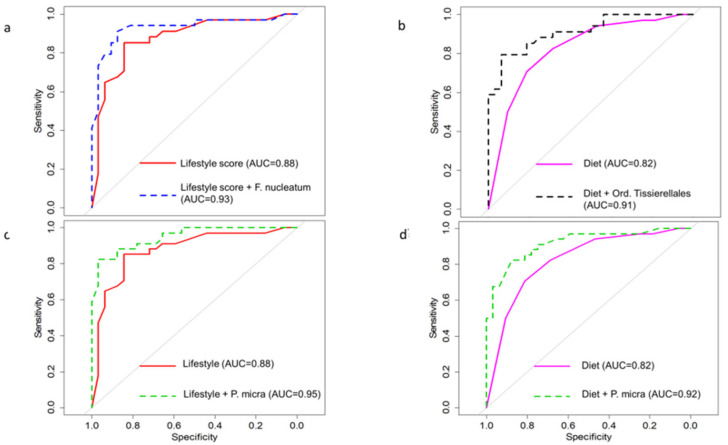
Results from multivariate logistic models: receiver operating characteristics (ROC) curves and area under the ROC curve (AUC) adding a taxa to a diet score or lifestyle score. (**a**) Lifestyle score + *F. nucleatum*; (**b**) diet + class *Tissierella*; (**c**) lifestyle + *Parvimonas micra*; (**d**) diet + *Parvimonas micra*.

As shown in Figure 6 and Table 5, by applying Lasso and multivariable logistic models, we found that the sole evaluation of a lifestyle risk score or a dietary score, together with one of the more represented and reproducible bacterial taxa (in particular, *F. nucleatum*, or *Parvimonas micra* species or class *Tissierella*), was sufficient to yield high cross-validation performance for all models (area under the receiver operating characteristics (ROC) curve (AUC) between 88% and 91%). The inclusion of serum biomarkers did not significantly increase AUC.

### 3.6. Microbiome Associated with CRC Prognostic Factors and Relapse

**Figure 7:**
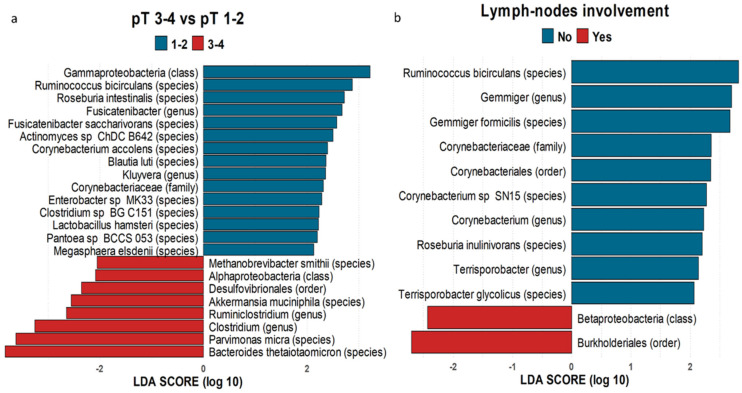
Bar plots of LDA score of 16s rRNA sequencing analysis by pT and pN. (**a**) Bar plot representing the significant differences between CRC tumor size 1–2 (blues) and 3–4 (red) subjects obtained by applying LEfSe on metabarcoding 16S data. Plots are generated by using ad hoc developed R script considering only the significant features. The bar length represents the LDA score (LDA score > 2). (**b**) Bar plot representing the significant differences between taxa and lymph node involvement (negative lymph node in blue and positive lymph node in red).

To investigate the association between microbiota composition and tumor staging, we compared the microbiome with tumor size (pathological T, pT) and lymph node involvement (pathological N, pN) using the LEfSe analysis. Comparisons were made between microbiome of pT1-2 and pT3-4 CRC patients and the microbiome of patients with or without involvement of regional lymph nodes (Figure 7). For tumor evaluation, we employed the TNM staging system, in which “T” is used to describe how deeply the primary tumor has grown into the bowel lining.

By performing this analysis, we found 15 taxa that were specific for pT1-2 patients and 8 taxa that were specific for pT3-4 patients. Interestingly, the *Ruminococcus bicirculans* species and the *Corynebacteriaceae* family were more abundant in pT1-2 patients (Figure 7a) and were also increased in patients with negative lymph nodes (Figure 7b). While 10 bacterial taxa were specifically enriched in patients without lymph node involvement, only *Betaproteobacteria* class and *Burkholderiales* order were specifically associated with patients with positive lymph nodes. The genera *Ruminiclostridium* and *Clostridium* were more specifically enriched in pT3-4 patients. *Parvimonas* and *Dialister* genera were very low among controls and the abundance increased among cases with worse prognosis (pT3-4 and N+; Supplementary Figure S3).

**Figure 8:**
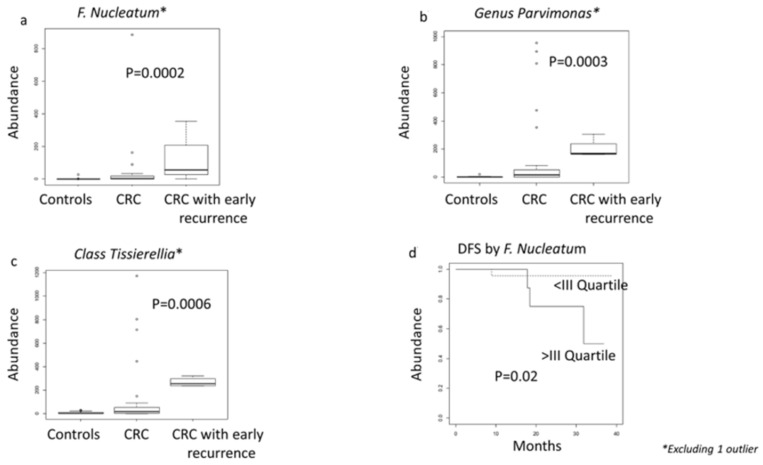
Box plots of taxa significantly associated with CRC status (**a**–**c**). Panel (**d**) Kaplan–Meier curves for disease-free survival *p*-value indicated in panel 8d was obtained from multivariate Cox regression models adjusting for lymph node status. * Excluding 1 outlier.

We also conducted an exploratory analysis at 29 months median follow-up and found four cases with cancer recurrence and five with adenomas. As shown in Figure 8a–c, abundance of *F. nucleatum*, *Parvimonas* species, and *Tissierella* class was significantly lower among controls, greater among cases with no recurrence, and very high in cases with cancer recurrence (Kruskall–Wallis *p* = 0.0002, *p* = 0.0003, *p* = 0.0006, respectively). After categorizing *F. nucleatum* into low and high abundance, considering the upper quartile of the distribution among cases, we found that high *F. nucleatum* was also significantly associated with time to relapse (log-rank *p* = 0.03; Figure 8d), with the association remaining statistically significant when adjusting the Cox proportional hazard model for lymph-node status (*p* = 0.02). Altogether, these data suggest that the microbiota composition plays a significant role throughout the tumorigenic process, including progression, and may influence prognosis.

## 4. Discussion

It is well established that several risk factors contribute to the development of CRC [2]. However, to date, there is an insufficient understanding of how the interplay between several factors such as vitamin D, dietary consumption, body mass index (BMI), fecal microbiota, inflammatory markers, and adipokines can affect CRC development and prognosis. In the present study, we integrated information on microbiome, diet, inflammatory serum biomarkers, and adipokines, which are all known risk factors for CRC. The results of our study did not only confirm that CRC correlates with complex host–environment interactions but showed also that the integration of lifestyle risk factors, serum biomarkers, and microbiome significantly improves our capability to discriminate healthy subjects from CRC patients.

We found that subjects who did not follow dietary guidelines from WCRF were at significantly higher risk to develop CRC. However, we also found that dietary habits not aligned with WCRF guidelines (high-risk diet) correlated with a general higher inflammatory status and reduction of zonulin. The latter is a component of the tight junctions, and its downregulation is associated with dysbiosis [33]. Accordingly, in these patients, we also identified an enrichment of several bacterial pathobionts, particularly *F. nucleatum*, which accelerates the onset of colonic tumors by driving the transition to a pro-inflammatory microenvironment [34,35] and *Clostridium ramosum*. The latter was shown to increase the expression of the glucose transporter 2 (Glut2) in jejunal mucosa and the fatty acid translocase (CD36) in ileal mucosa animal studies using high-fat diet and may contribute to increased absorption of carbohydrates and fat [36]. Conversely, subjects following WCRF guidelines showed a significant enrichment of several bacteria that contribute to gut homeostasis, such as those belonging to the genus *Anaerostipes*, which produce butyrate, a compound with anti-inflammatory and antineoplastic properties [37,38].

These findings are in agreement with a recent study showing that microbiota functional pathways can discriminate healthy subjects from CRC patients and that microbiota-mediated metabolic activities can contribute to CRC development via production of pro-carcinogenic compounds such as polyamine [6,39,40]. Interestingly, we also found an inverse association between CRC risk and high fatty fish consumption but not with other types of fish. These results are in agreement with a recent meta-analysis [41,42], showing that fish consumption is inversely associated with colorectal cancer. Polyunsaturated fatty acids in fish have the capacity to regulate cell proliferation and apoptosis in human colorectal cancer cell lines. Interestingly, fish consumption had no impact on apoptosis induction ex vivo [43]. Moreover, fatty fish is a source of dietary vitamin D, and high consumption can increase serum 25(OH) vitamin D [44]. Considering the anti-inflammatory and antitumorigenic properties of vitamin D, this can explain why fatty fish consumption in our data and in published reports is associated to lower CRC risk.

A recent meta-analysis [45] assessed the effect of the gut microbiome on the relationship between obesity and increased CRC risk. The investigators reported that the association between BMI and CRC risk was only slightly attenuated when several CRC-associated taxa were added to the analytic model, indicating a weak effect of the microbiome as mediator of obesity on CRC. In our mediation analysis, BMI and adiponectin levels were not found to be significant mediators of diet. However, the *Bifidobacteria*/*Escherichia* genera ratio in the mediation analysis showed that subjects exposed to a high-risk diet have a significantly decreased CRC risk with increasing levels of *Bifidobacteria* over *Escherichia*. The inverse ratio of *Bifidobacterium* to *Escherichia coli* is a dysbiosis associated with colorectal cancer. In particular the number of *Bifidobacterium* decreased significantly in CRC, while *Escherichia* increased [46].

Altogether these findings not only support that diet, lifestyle, and gut microbiota interact with inflammation and CRC risk, but the microbiome may still be able protect the individual from CRC risk independently on the type of diet.

An international panel of experts published recommendations for CRC screening suggesting that CRC screening should be performed in subjects with an estimated 3% or higher CRC risk within 15 years [47]. These recommendations are based on a prediction model characterized by an area under the receiver operating curve (AUC) of about 85% in the development cohort and 66–70% in the external validation cohort [47].

In the recently published pooled analysis [6], the authors identified microbial signatures, trained on multiple datasets, that have consistently high accuracy in both training and independent validation cohorts (average AUC = 84%). In our study, by considering a single microbiome reproducible biomarker and a lifestyle risk score, we could predict CRC cancer risk with very high sensitivity and specificity (AUC > 90%). Thanks to the technology improvement, molecular tests are also becoming affordable and, for this reason, according to risk score (e.g., algorithm risk evaluation, personal, and/or familial history), fecal microbiome signature could provide an alternative or a second level test for personalized screening program.

The major limitation of our study is the relatively small sample size and consequent reduced statistical power, which we partially compensated for by analyzing a high number of variables. Even if we did not have a validation set, when we performed a leave-one-out cross validation, the AUC remained greater than 80%. Another limitation was that the analyses were conducted at a single time point and therefore reverse causation bias cannot be ruled out. Furthermore, cases and controls were not balanced for family history; indeed, among CRC cases, we had less subjects with family history compared to controls. This difference was due to the inclusion criteria for the controls—a recent clean colonoscopy was required to not include subjects with adenomas or very initial cancer among controls. However, this difference was not significant in multivariable analyses after adjusting for other confounders. Lastly, we administered a short questionnaire to measure dietary consumption, and even if it is a good surrogate of the dietary intake [27], it represents a limitation as this tool is not error-free [48].

## 5. Conclusions

The present study suggests that diet, microbiome, vitamin D, markers of inflammation, and adipokines are strongly connected in a complex network, and the unbalance of one or more factors may contribute to cancer incidence and prognosis. In particular, a diet poor in fatty fish and rich in carbohydrates may be associated with CRC risk, but microbiome may mediate this effect. Additional studies are needed to develop a more inclusive method to improve preventive strategies, including screening tools, risk assessment, and stratification toward a more personalized surveillance. Furthermore, these are key factors for hypothesis-driven mechanistic studies to develop intervention for cancer preventive medicine.

## Figures and Tables

**Table 1:** Descriptive characteristics and biomarkers of colorectal cancer patients (*n* = 34) and controls (*n* = 32).

		CRC (*N*, %)	Controls (*N*, %)	Total (*N*, %)	*p*-Value
Sex	Females	10 (29.4)	14 (43.7)	24 (36.4)	0.23
	Males	24 (70.6)	18 (56.3)	42 (63.6)	
Age	≤60 years	18 (52.9)	20 (62.5)	38 (57.6)	0.43
	>60 years	16 (47.1)	12 (36.5)	28 (42.4)	
BMI	≤25	11 (33.3)	20 (62.5)	31 (47.7)	0.02
	>25	22 (66.7)	12 (37.5)	34 (52.3)	
Regular physical activity	No	20 (58.8)	8 (25.0)	28 (42.4)	0.006
	Yes	14 (42.2)	24 (75.0)	38 (57.6)	
Regular alcohol consumption	No	5 (14.7)	15 (46.9)	20 (30.3)	0.005
	Yes	29 (85.3)	17 (53.1)	46 (69.7)	
Colon cancer family history	No	25 (73.5)	16 (50.0)	41 (62.1)	0.05
	Yes	9 (26.5)	16 (50.0)	25 (37.9)	
Smoking	Never	12 (35.3)	14 (75.0)	36 (54.5)	0.005
	Current	9 (26.5)	3 (9.4)	12 (18.2)	
	Former	13 (38.2)	5 (15.6)	18 (27.3)	

CRC, colorectal cancer; BMI, body mass index. *p*-values were obtained with chi-squared test.

**Table 2:** Descriptive statistics of serum biomarkers of colorectal cancer patients and controls.

			CRC			Controls		
		Median	Lower Quartile	Upper Quartile	Median	Lower Quartile	Upper Quartile	*p*-Values
25-OHD (ng/mL) ^1^		19.8	11.2	25.1	23.4	16.1	31.4	0.12
VDBP (µg/mL)		235	166	295	249	209	309.5	0.58
Zonulin (ng/mL)		119	74	178	109	54	315	0.94
IGFII (ng/mL)		671	578	769	695	614	806	0.41
IGFBP3 (µg/mL)		2.17	1.95	2.59	2.36	2.16	2.64	0.09
CRP (mg/dL)		0.23	0.12	0.39	0.10	0.05	0.20	0.01
Adiponectin (µg/mL)		4.87	3.41	9.48	7.77	6.23	12.39	0.03
Leptin (ng/mL)		6.56	4.25	14.15	6.71	5.19	15.43	0.67
		N. (%)			N. (%)			
Vitamin D (ng/mL) ^1^	Sufficient	24 (70.6)			29 (90.6)			0.04
	Deficient	10 (29.4)			3 (9.4)			
hs-CRP (mg/dL) ^2^	≤0.1	7 (20.6)			16 (50)			0.012
	>0.1	27 (79.4)			16 (50)			
Adiponectin (µg/mL) ^3^	≤6	20 (58.8)			7 (21.9)			0.002
	>6	14 (41.2)			25 (78.1)			
IL-6 (pg/mL) ^4^	≤4	25 (73.5)			30 (93.8)			0.03
	>4	9 (26.5)			2 (6.3)			

Differences between median values were assessed with Wilcoxon rank tests and differences in frequencies with chi-squared tests.^1^ Vitamin D deficiency is defined relative to the season: <20 ng/mL in summer/autumn and <10 ng/mL in winter/spring.^2^ Cut-off point chosen on the basis of median value of controls. ^3^ Cut-off point chosen on the basis of first quartile among controls.^4^ Cut-off point chosen on the basis of the literature.

**Table 3:** Frequencies of colorectal cancer patients and controls by mutation status of polymorphisms.

VDR, GC, and CYP SNPs		CRC *n* = 34(%)	Controls *n* = 32 (%)	Total *n* = 66 (%)	*p*-Value
*Fok1 rs2228570* (A > G) *FokI*	GG (*FF*) or GA (*Ff*)	27 (79.4)	31 (96.9)	58 (87.9)	0.03
(A = rare nucleotide)	AA (*ff*)	7 (20.6)	1 (3.1)	8 (12.1)	
*Bsm1 rs1544410* (C > T) *BsmI*	CC (*bb*) or CT (*Bb*)	31 (91.2)	27 (84.4)	58 (87.9)	0.39
(T = rare nucleotide)	TT (*BB*)	3 (8.8)	5 (15.6)	8 (12.1)	
*Taq1 rs731236* (A > G) *TaqI*	AA (*TT*) or AG (*Tt*)	32 (94)	27 (84)	58 (89)	0.20
(G = rare nucleotide)	GG (*tt*)	2 (6)	5 (16)	7 (11)	
*Apa1 rs7975232* (C > A) *ApaI*	AA (*AA*) or AC (*Aa*)	27 (79.4)	25 (78.1)	52 (78.8)	0.9
(C = rare nucleotide)	CC (*aa*)	7 (20.6)	7 (21.9)	14 (21.2)	
*GC rs2282679* (T > G)	TT or TG	31 (91.2)	31 (96.9)	62 (93.9)	0.33
(G = rare allele)	GG	3 (8.8)	1 (3.1)	4 (6.1)	
*GC rs4588* (G > T)	GG or GT	31 (91.2)	31 (96.9)	62 (93.9)	0.33
(T = rare nucleotide)	TT	3 (8.8)	1 (3.1)	4 (6.1)	
*GC rs7041* (A > C)	CC or CA	27 (79.4)	28 (87.5)	55 (83.3)	0.38
(A = rare nucleotide)	AA	7 (20.6)	4 (12.5)	11 (16.7)	
*CYP24A1 rs6013897* (T > A)	TT or TA	29 (85.3)	32 (100)	61 (92.4)	0.02
(A = rare nucleotide)	AA	5 (14.7)	0 (0.0)	5 (7.6)	
*CYP27B1 rs10877012* (G > T)	GG or GT	31 (91.2)	29 (90.6)	60 (90.9)	0.93
(T = rare nucleotide)	TT	3 (8.8)	3 (9.4)	6 (9.1)	
CYP2R1 rs10741657 (A > G)	GG or GA	31 (91.2)	32 (100)	63 (95.5)	0.09
(A = rare nucleotide)	AA	3 (8.8)	0 (0)	3 (4.5)	

VDR, vitamin D receptor; GC, Vitamin D Binding Protein gene; CYP, cytochrome P450; SNPs Single Nucleotide Polymorphism; *p*-values were obtained with chi-squared test and Fisher’s exact test.

**Table 4:** Multivariable logistic models: diet and risk factors associated with CRC.

	Lifestyle Risk Score	OR	Lower 95% CI	Upper 95% CI	*p*-Values
Risk factors	Regular physical activity	0.28	0.08	0.99	0.049
	Ever smoking	3.21	0.85	12.14	0.086
	High alcohol	6.20	1.27	30.20	0.024
Diet	High sweets and cakes	4.31	1.02	18.28	0.048
	Low fatty fish and highcereals/carbohydrates ^2^	5.88	1.49	25.0	0.011
	WCRF score ^1^	0.23	0.08	0.67	0.007

*p*-values were obtained from multivariable logistic models. ^1^ WCRF score: adherent if BMI < 25, high physical activity and a healthy diet (high consumption of fruit and vegetables, or low consumption of meat or sweets, cakes, and pastries). ^2^ Low fatty fish and high cereals/carbohydrates: Low fatty fish (salmon, herring, mackerel) less than twice a week and high cereals (pasta, rice, and bread) at least once a day.

**Table 5:** Performance of multivariable logistic models including microbiome and serum biomarkers and dietary and lifestyle^¥^ risk scores.

			Cross Validation
	*p*-Value	AUC	AUC (95% CI)
Lifestyle score	0.0002	93%	91% (83–99)
*F. nucleatum*	0.006		
Lifestyle score	0.0002	95%	91% (86–98)
*Parvimonas micra*	0.003		
Dietary score	0.0004	92%	88% (80–96)
*Parvimonas micra*	0.003		
Dietary score	0.0004	91%	89% (81–96)
*Tissierella*	0.001		

AUC: area under the ROC curve. Taxa were introduced in the model considering the log transformation. ^¥^ Lifestyle risk score includes physical activity, alcohol and smoking, and significant dietary factors. Dietary score is obtained from the estimates of the multivariable logistic model of Table 3, considering significant dietary factors, adjusted for physical activity, alcohol, and smoking. *p*-values were obtained with a chi-squared test.

## Data Availability

Nucleotide sequences are available in the Sequence Read Archive under accession no. SRP136711. MetaPhlAn2 and HUMANn2 profiles were also added to the curated Metagenomic-Data R package27, along with their corresponding metadata. European Nucleotide Archive under the study identifier PRJEB27928. Data on serum biomarkers and dietary information are available from the corresponding author on reasonable request.

## Supplementary Materials

The following are available online at https://www.mdpi.com/2072-6643/13/2/363/s1: Sup-plementary Figure S1. Bar chart: percentages of colorectal (CRC) patients by vitamin D level and Parvimo-nas genus. Figure S2. Triplot of Canonical Correspondence Analysis, Figure S3. Association of microbiota with colorectal cancer (CRC) prognostic factors, Table S1. Descriptive statistics of 25-OHD levels (ng/mL) by seasons and colorectal cancer sta-tus, Table S2. Multivariable logistic models including microbiome taxa and serum biomarkers, Table S3. Descriptive statistics of 25-OHD levels (ng/mL) by consumption of fatty fish (dose: 150 gr) and colorectal cancer status, Table S4. Food intake frequen-cies for colorectal cancer patients and controls.

## Abbreviations

25(OH)D	25-Hydroxyvitamin D
AUC	Area under the curve
BMI	Body mass index
CRC	Colorectal cancer
F.	Fusobacterium
hs-CRP	High-sensitivity C-reactive protein
IBD	Inflammatory bowel diseases
IL-6	Interleukin-6
LDA	Linear discriminant analysis
LEfSe	Linear discriminant analysis effect size
OTUs	Operational taxonomic units
ROC	Receiver operating characteristics
ORs	Odds ratios
AUC	Area under the ROC curve
VDBP	Vitamin D-binding protein
CCA	Canonical correspondence analysis
WCRF	World Cancer Research Fund International
FDR	False discovery rate
