# Supplementary material for: Microbiome as Mediator of Diet on Colorectal Cancer Risk: The Role of Vitamin D, Markers of Inflammation and Adipokines
Source: Nutrients. 2021 Jan 25;13(2):363. doi: 10.3390/nu13020363 (PMC7911673; doi:10.3390/nu13020363)
Supplement: Supplementary file 1 [file nutrients-13-00363-s001.pdf]

SUPPLEMENTARY INFORMATION

SUPPLEMENTARY MATERIALS AND METHODS

Bioinformatic and statistical methods

Bioinformatic analysis for comparison between CRC and healthy controls

For microbiome analysis, the taxonomic annotation of raw Illumina MiSeq reads was performed using the pipeline BioMaS [1]. In particular: (i) the overlapping Paired End (PE) reads were merged into consensus sequences using pair-end read merger (PEAR) [2] and sequences shorter than 50nt were removed. Non-overlapping PE reads were further denoised by removing low-quality regions (quality-score threshold equal to 25) and discarding PE reads containing sequences shorter than 50nt by using Trim-Galore; (ii) both consensus and unmerged PE reads were mapped on the 11.5 release of the RDP II database [3, 4] using Bowtie2 [5]; (iii) to obtain the taxonomic classification, mapping data were filtered according to two parameters: identity percentage (≥90%) and query coverage (≥ 70%). In particular, sequences matching on RDP II with an identity percentage of at least 97% were directed to species classification [6], while the others were classified at higher taxonomic levels. The NCBI taxonomy was used as reference taxonomy.

To infer the alpha-diversity parameters, raw Illumina sequences in OTUs (Operational Taxonomic Units) were filtered by applying QIIME [7] In particular: (i) adaptor trimming: Illumina Nextera adaptor was removed by applying trim galore; (ii) PE reads were merged by applying PEAR [2]; (iii) OTU definition was achieved by applying the QIIME open-picking procedure (refer-

ence database and taxonomy: greengenes 13.8); (iv) chimeric sequences were removed using Chimera-Slayer [8]. The OTU table was re-generated by excluding chimeric OTUs and normalized by rarefaction.

12.5 million PE reads were produced in the 16S rRNA analysis. The mean number of PE reads per sample was about 187,000. About 97.5% of the sequences were taxonomically annotated using BioMaS. In particular, 89.5% and 68.2% of the sequences were taxonomically annotated at genus and species rank, respectively. In total, 744 operational taxonomic units (OTUs) were detected using 97% similarity threshold. The total number of sequences represents ranges from 115,437 to 204,779 sequences (Median 169,084, Average 168,265). Taxonomic data were summarized at phylum, class, order, family, genus and species level, normalized by applying DESeq2 [9] and analyzed by using Linear Discriminant Analysis (LDA) effect size (LEfSe) [10]. LEfSe results were plotted using an in-house developed R script and GraPhlAn [10]. Univariate analysis on a per dataset basis was performed using LEfSe to identify features that were statistically different among groups and estimate their effect size. All p-values were set at 0.05, two-sided, adjusting for False Discovery Rate (FDR). We also presented results of multivariable logistic models: taxa significantly associated with CRC status, after adjustment for FDR and for significant confounding factors.

To better understand microbiota composition and its role in the CRC carcinogenesis we analyzed also microbiota species and pathways from shotgun metagenomics. Shotgun data were missing for 2 CRC patients and 5 controls. Quantitative taxonomic profiling was performed using MetaPhlAn2 [11], whereas HUMANn2 [12] was used to profile pathway and gene family abundances. The generated profiles are available through the curated MetagenomicData R package [13].

Statistical analysis to investigate independent role of microbiome and interactive factors

Overall we applied classical statistical multivariate methods measuring significant abundances, adjusted only for FDR (such as Linear Discriminant Analysis effect size [10]), and multivariate methods adjusted also for confounders, which integrate different types of dataset (such as multivariable logistic models, Data Integration Analysis for Biomarker Discovery and priority-LASSO hierarchical approaches) [14]. Furthermore, we compared results obtained with 16S and shotgun data.

Information on lifestyle risk factors, diet and serum biomarkers are summarized with descriptive characteristics and compared between cases and controls. Median and interquartile range for continuous variables and absolute and relative frequencies for categorical variables are presented for cases and controls. Two-independent samples Wilcoxon test, Chi-square (Fisher exact test for sparse data) were used to assess differences between groups. We employed linear regression models after verifying normal distribution of residuals from full models. We graphically checked normal probability plots, which are designed to specifically test for the assumption of normality, Q Q plots, which compare our data to data from a distribution with known normality, and also the histograms of residuals, which are useful to investigates whether data meets assumption.

Serum biomarkers were investigated as continuous variables and categorical variables. 25-OHD levels are subjected to great seasonal variation and vitamin D supplementation is usually prescribed taking into account the levels of 25-OHD in winter [15-17]. Therefore, the comparison of 25-OHD in subjects enrolled in summer with 25-OHD in subjects enrolled in winter would have introduced a bias. To avoid this bias, we decided to consider a cut-off value for 25-OHD based on the season of blood collection (**Supplementary Table S1**), we defined the following cut-off points for vitamin D deficiency: values below 10 ng/ml in winter, spring and initial summer (from November to June)

and below 20 ng/ml in late summer and autumn [18]. The cut-off point for hs-CRP was chosen based on the median value of controls, the cut-off point for adiponectin was chosen based on first quartile among controls. The cut-off point chosen for IL-6 was based on studies showing that IL-6 was associated with bone loss and resorption [19].

To develop the World Cancer Research Fund International (WCRF) score, we took into account the dietary recommendations for cancer prevention published by WCRF/American Institute for Cancer Research [20, 21]. Subjects were considered adherent if their BMI was lower than 25, they declared high physical activity and a healthy diet (high consumption of fruit and vegetable or low meat sweets, cakes and pastries consumption). We also build two dietary scores: one was a categorical variable and the other was a continuous variable obtained from the multivariable logistic model, including significant risk factors and confounders.

Non-parametric Wilcoxon-Ranks and Kruskall-Wallis tests were used to investigate differences in abundance of normalized taxa among CRC and controls as shown in the box plots.

The network inference was based on Spearman's rank Correlation Coefficient and plotted by using the graph R package [22] including all the following continuous variables: serum biomarkers, species (normalized data) that were significantly associated with CRC in the sPLS-DA analysis (adjusting for FDR), BMI and a dietary score obtained from a multivariable logistic model, with cases vs controls as endpoint, adjusted for confounders. Correlation values were computed on pairwise complete observation, by adopting the *cor* function of *stats* R package (R 3.4 version, https://www.r-project.org/) and choosing as a measure the Spearman coefficient, a non-parametric measure of rank correlation.

We used Canonical Correspondence Analysis (CCA) [23] to further explore possible clinical inflammatory markers associated with salivary microbiota community structure. CCA is an unsupervised method aimed to identify the linear combinations of two sets of variables that are maximally correlated with each other and provides an important tool to summarize the overall dependency structures between two sets of variables such as taxa and serum biomarkers. We carried out the analysis on the log transformation of taxa with the highest association with cases and we obtained a graphical representation of the first two components (triplot) for cases and controls.

We combined microbiome normalized data with serum biomarkers, BMI and diet variables by using the Data Integration Analysis for Biomarker Discovery [24]. DIABLO is a recent framework of the mixOmics R package for the integration of different dataset in supervised analysis [25]. This allowed us to discriminate between the CRC and healthy controls by a block sPLS-DA supervised model.

A first step includes the creation of a priori knowledge design matrix for determining which dataset blocks should be maximally correlated between components. A correlation of 0.2 was imposed among the above mentioned dataset blocks. Then the best number of components was tuned, by running a 10-fold cross-validation repeated 10 times DIABLO model without selecting any variables. A grid of values indicating the range (min-max) of variables, that might be selected among blocks, was provided. In particular, a range from 5 to 11 variables was adopted and the optimum number of variables, to select in each block, was chosen for the first two components. Finally, the final sparse Partial Least Square – Differential Analysis (sPLS-DA) model was fit (10-fold cross-validation and 100 repeats) by using the tuning setting found in previous steps.

A Heatmap plot was generated to graphically selecting the most discriminative species using the first and second component loading vectors.Heatmap plot was generated by performing a sparse Partial Least Square – Differential Analysis (sPLS-DA) (10-fold cross-validation and 100 repeats) and selecting the most discriminative species using the first and second component loading vectors.

Mediation analysis

To estimate the role of *microbiome* as mediator of dietary factors on CRC development, we performed a mediation analysis based on a counterfactual framework approach [26, 27]. We decomposed the total effect (TE) of diet on CRC into a natural direct effect (NDE) and a natural indirect effect (NIE) acting on cancer through microbiome, considering alcohol consumption and physical activity as possible confounders. The NDE of *diet* was estimated by comparing the effect of a high risk diet (low fat fish and high cereal/carbohydrates consumption) versus the effect of a healthy diet (high fat fish and low cereal/carbohydrates consumption) on CRC, having the microbiome set to the value it would naturally have under the non-risk condition. The NIE was estimated by considering the exposure to risk and comparing the effect of the microbiome under a high risk diet versus the effect of the microbiome under a low risk diet on CRC. In agreement with previous publications [28, 29], microbiome was evaluated considering the ratios of *Bifidobacteria* to *Escherichia* genera and *Firmicutes* to *Bacteroides* phyla, which have been shown to be modified with obesity [30]. Both ratios were log-transformed, adding 1 unit, and modelled as mediators with linear regression models, whereas ORs with 95% CIs were obtained for NIE using unconditional logistic regression models adjusting

for alcohol and physical activity. For comparison, we evaluated also BMI and adiponectin as mediators of diet.

The formulas used to calculate each effect are as follows.

Let Y be the binary outcome (CRC status), A the exposure (diet), M the mediator variable (microbiome) and C a set of multiple confounders (alcohol and physical activity).

The outcome Y was modelled using logistic regression as follows:

$logit\{P(Y = 1|a, m, c)\} = \theta_0 + \theta_1 a + \theta_2 m + \theta_3 am + \theta_4{}'c$where c is the vector of confounders.

The mediator M was modelled using linear regression as follows:

$$M = \beta_0 + \beta_1 a + \beta_2{}'c$$

where c is the vector of confounders.

Provided that the assumption that the outcome Y is rare holds, we derived NDE and NIE on the Odds Ratio scale as following:

$$log\{OR^{NDE}\} = \{\theta_1 + \theta_3(\beta_0 + \beta_1 a^* + \beta_2' c + \theta_2 \sigma^2)\}(a - a^*) + 0.5\theta_3^2 \sigma^2 (a - a^2)$$

where σ^2 is the variance of the error term in the regression model on mediator M. For the binary exposure A, the two levels being compared are a^*=0 and a=1.

Thus, the NDE provides an estimate of how much the outcome Y would change if the exposure A were set at level a=1 versus level a^*=0, having the mediator set to level it would naturally have in the absence of exposure.

$$log\{OR^{NIE}\} = \theta_2 \beta_1 (a - a^*)$$

Thus, the NIE estimates how much the outcome Y would change if the exposure *A* was set to level a=1, but the mediation *M* changes from the level it would have if a*=0 to the level it would take if a=1.

Prediction of CRC

Abundances were log-transformed (after adding a pseudo-count of 1 to avoid non-finite values resulting from log(0)) and priority-LASSO (hierarchical approach) [14] unconditional logistic regression model was applied as well as backward and forward selection of variables. We used priority-LASSO models with the following block structure, according to their level of priority: "Serum biomarkers" (25-OHD, adiponectin, hs-CRP etc.) and "metagenomic data". The lambda parameter, i.e. regularization strength was selected as the minimum value that maximizes the area under the Receiver Operating Characteristics curve (ROC). A clustering analysis was applied to group similar microbial communities, using Spearman correlation, in order to identify taxa representative of the variability but not closely correlated with others. The number of clusters was established adopting the R package "NbClust", choosing the "Calinski and Harabasz Index" as score (maximum value). We then integrated the clustering results together with the variables selected by priority-LASSO models and PLS-DA in order to choose significant independent biomarkers for the multivariable logistic models. Models were then evaluated by calculating the area under the ROC curve (AUC). Leave-one-out cross-validation of the multivariable models was also applied and the correspondent cross-validated AUC was calculated.

Subgroup analyses to evaluate associations with prognostic factors and CRC recurrence

We evaluated differences in abundance of microbiome biomarkers among controls and cases grouped considering pT (pT1-2 vs pT3-4), pN (pN+, i.e. evidence of lymph-node involvement, and pN0) and early recurrence. Non-parametric Wilcoxon rank and Kruskall-Wallis tests were used.

F. nucleatum was categorised in quartiles to verify whether high abundance was associated with time to relapse in cases, calculated from diagnosis to first cancer relapse or last follow-up. We calculated Kaplan-Meier curves for disease free survival. Log-rank tests were evaluated to investigate differences between curves. All statistical tests were two-sided. Statistical analyses were performed using the SAS statistical software (version 9.02 for Windows) and R 3.4 version.

SUPPLEMENTARY FIGURE LEGENDS

Supplementary Figure S1

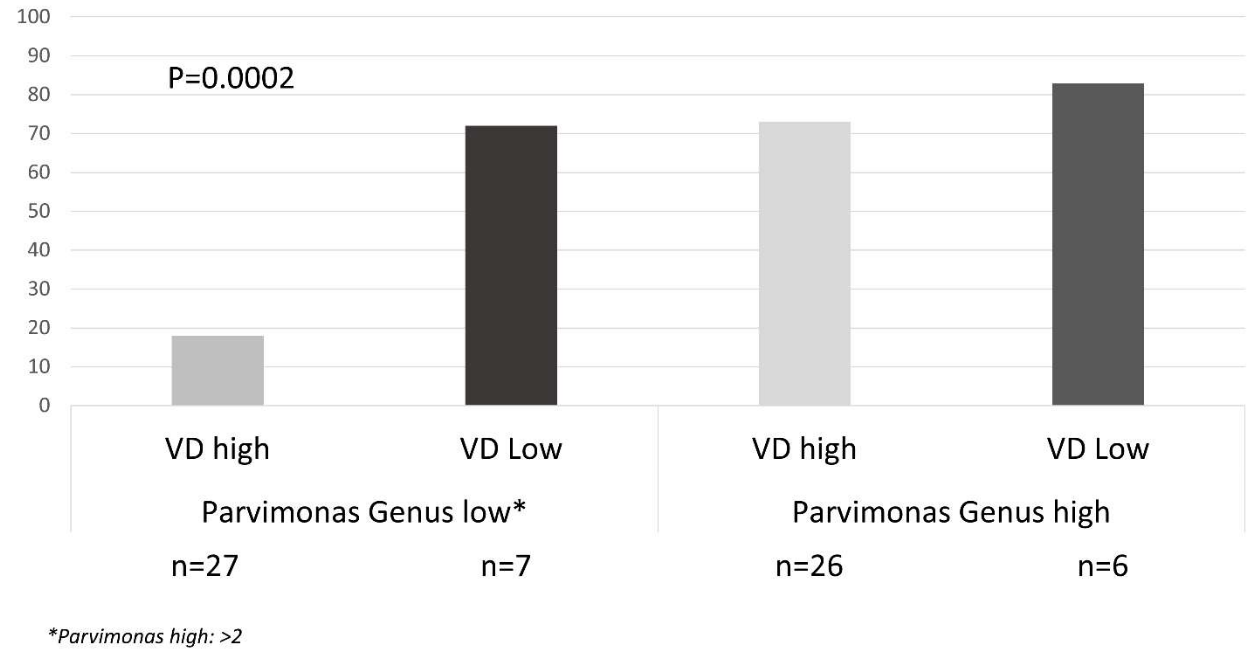

Supplementary Figure S1. Bar chart: percentages of colorectal (CRC) patients by vitamin D level and *Parvimonas genus*.

Supplementary Figure S2

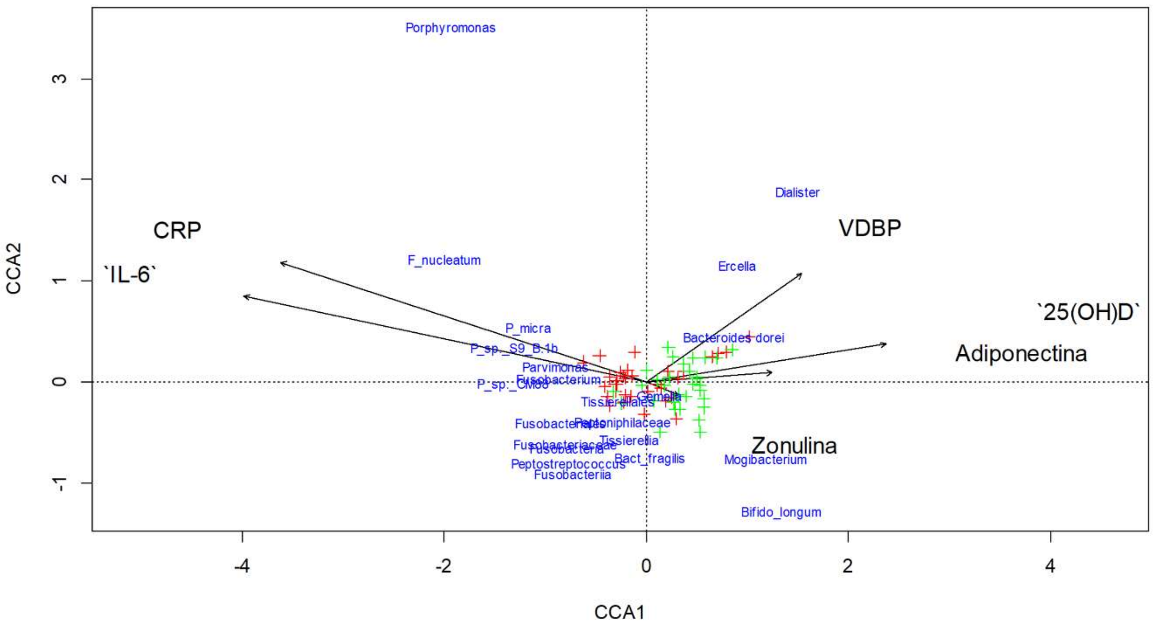

Supplementary Figure S2. Triplot of Canonical Correspondence Analysis.

Arrows indicate the direction and magnitude of the serum biomarkers, systemic inflammatory markers associated with bacterial community structures. Healthy controls are in green and colorectal cancer cases in red.

Supplementary Figure S3

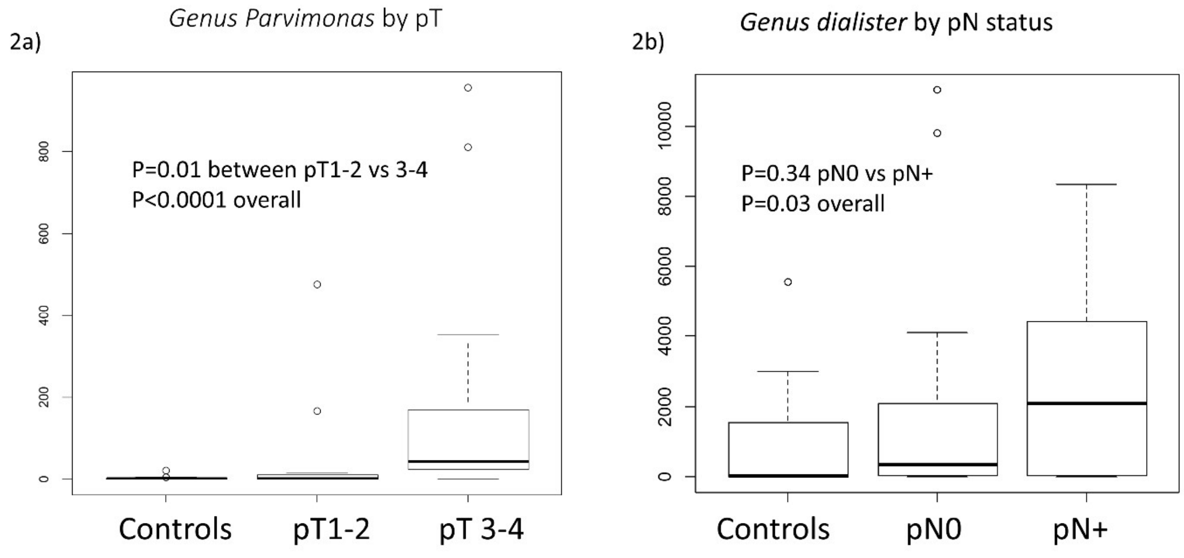

Supplementary Figure S3. Association of microbiota with colorectal cancer (CRC) prognostic factors.

On the left, bar-plot representing the association of *Genus Parvimonas* in CRC patients with pathological tumor (pT) 1-2, with pT3-4 and controls. On the right, bar-plot representing the association of *Genus dialister* in CRC patients without lymph-node (pN0), with lymph-node involvement (pN+) and controls.

SUPPLEMENTARY TABLES

Supplementary Table S1. Descriptive statistics of 25-OHD levels (ng/mL) by seasons and colorectal cancer status

Season	Status	N	Median	Mean	Lower Quartile	Upper Quartile
Summer-Autumn	Cases	12	22.9	24.4	17.1	34.4
	Controls	14	30.8	31.4	25.5	35.3
Winter	Cases	8	23.5	20.9	15.9	27.4
	Controls	5	18.8	19.1	13.9	19.6
Spring	Cases	14	13.1	14.2	7.3	21.6
	Controls	13	17.9	16.6	12.7	22.6

Supplementary Table S2. Multivariable logistic models including microbiome taxa and serum biomarkers

	P-values	AUC	Cross validation
Vitamin D low	0.017	91%	81%
F. Nucleatum	0.036		
genus *Dialister*	0.047		
Adiponectin low	0.001		
Genus *Fusobacterium*	0.021	90%	85%
25-OHD	0.042		
adiponectin low	0.002		

AUC, area under the curve

Supplementary Table S3. Descriptive statistics of 25-OHD levels (ng/mL) by consumption of fatty fish (dose: 150 gr) and colorectal cancer status

Frequency of fatty fish consumption	Status	N	Median	Lower Quartile	Upper Quartile
Rarely (once/twice a month)	Cases	17	19.3	12.8	24.5
	Controls	10	18.5	13	23.4
Once a week	Cases	13	15.2	9.7	23.8
	Controls	11	24.5	14.2	30
Two-three times a week	Cases	4	31.05	17.7	34.4
	Controls	11	26	17.9	35.3

Supplementary Table S4. Food intake frequencies for colorectal cancer patients and controls

Foods consumptions	Categories	CRC (N, %)	Controls (N, %)	Total (N, %)	P-value
Dairy products (milk/cheese/yogurt)	Not every day	5 (14.7)	8 (25.0)	13 (19.7)	0.29
	Once a day	29 (85.3)	24 (75.0)	53 (80.3)	
Pasta, rice and bread	Not every day	11 (32.4)	23 (71.9)	34 (51.5)	0.001
	Once a day	23 (67.6)	9 (28.1)	32 (48.5)	
Fruit and vegetables	Not every day	6 (17.6)	5 (15.6)	11 (16.7)	0.83
	Once a day	28 (82.4)	27 (84.4)	55 (83.3)	
Meat or processed meat	At most once a week	4 (11.8)	4 (12.5)	8 (12.1)	0.93
	At least twice a week	34 (88.2)	28 (87.5)	58 (87.9)	
Eggs	Rarely	9 (26.5)	9 (28.1)	18 (27.3)	0.49
	Once a week	15 (44.1)	17 (53.1)	32 (48.5)	
	2-3 times a week	10 (29.4)	6 (18.8)	16 (24.2)	
Fatty fish (salmon, herring, mackerel)	Rarely	17 (50)	10 (31.3)	27 (40.9)	0.03
	Once a week	13 (38.2)	11 (34.4)	24 (36.4)	
	2-3 times a week	4 (11.8)	11 (34.4)	15 (22.7)	
Fish (other)	Rarely	10 (29.4)	6 (18.8)	16 (24.2)	0.57
	Once a week	15 (44.1)	14 (43.8)	29 (43.9)	
	2-3 times a week	8 (23.5)	10 (31.3)	18 (27.3)	
Sweet/cakes/chocolate	≤1 a week	8 (23.5)	14 (43.8)	22 (33.3)	0.08
	≥2 times a week	26 (76.5)	18 (56.3)	44 (66.7)	

P-values were obtained with Chi-squared test
Meat consumption includes any type of meat (white and red), including processed meat and liver consumption.
CRC, colorectal cancer

Reference List

[1]Fosso B, Santamaria M, Marzano M, et al. BioMaS: a modular pipeline for Bioinformatic analysis of Metagenomic AmpliconS. *BMC Bioinformatics* 2015 Jul 1,**16**, 203.

[2]Zhang J, Kobert K, Flouri T, Stamatakis A. PEAR: a fast and accurate Illumina Paired-End reAd mergeR. *Bioinformatics* 2014 Mar 1,**30**(5), 614-620.

[3]Cole JR, Wang Q, Cardenas E, et al. The Ribosomal Database Project: improved alignments and new tools for rRNA analysis. *Nucleic Acids Res* 2009 Jan,**37**(Database issue), D141-D145.

[4]Cole JR, Wang Q, Fish JA, et al. Ribosomal Database Project: data and tools for high throughput rRNA analysis. *Nucleic Acids Res* 2014 Jan,**42**(Database issue), D633-D642.

[5]Langmead B, Salzberg SL. Fast gapped-read alignment with Bowtie 2. *Nat Methods* 2012 Mar 4,**9**(4), 357-359.

[6]Mende DR, Sunagawa S, Zeller G, Bork P. Accurate and universal delineation of prokaryotic species. *Nat Methods* 2013 Sep,**10**(9), 881-884.

[7]Caporaso JG, Kuczynski J, Stombaugh J, et al. QIIME allows analysis of high-throughput community sequencing data. *Nat Methods* 2010 May,**7**(5), 335-336.

[8]Haas BJ, Gevers D, Earl AM, et al. Chimeric 16S rRNA sequence formation and detection in Sanger and 454-pyrosequenced PCR amplicons. *Genome Res* 2011 Mar,**21**(3), 494-504.

[9]Love MI, Huber W, Anders S. Moderated estimation of fold change and dispersion for RNA-seq data with DESeq2. *Genome Biol* 2014,**15**(12), 550.

[10] Segata N, Izard J, Waldron L, et al. Metagenomic biomarker discovery and explanation. *Genome Biol* 2011 Jun 24,**12**(6), R60.

[11] Truong DT, Franzosa EA, Tickle TL, et al. MetaPhlAn2 for enhanced metagenomic taxonomic profiling. *Nat Methods* 2015 Oct,**12**(10), 902-903.

[12] Franzosa EA, McIver LJ, Rahnavard G, et al. Species-level functional profiling of metagenomes and metatranscriptomes. *Nat Methods* 2018 Nov,**15**(11), 962-968.

[13] Pasolli E, Schiffer L, Manghi P, et al. Accessible, curated metagenomic data through ExperimentHub. *Nat Methods* 2017 Oct 31,**14**(11), 1023-1024.

[14] Klau S, Jurinovic V, Hornung R, Herold T, Boulesteix AL. Priority-Lasso: a simple hierarchical approach to the prediction of clinical outcome using multi-omics data. *BMC Bioinformatics* 2018 Sep 12,**19**(1), 322.

[15] Docio S, Riancho JA, PÃ©rez A, Olmos JM, Amado JA, GonzÃ¡lez-MacÃ-as J. Seasonal deficiency of vitamin D in children: a potential target for osteoporosis-preventing strategies? *J Bone Miner Res* 1998 Apr,**13**(4), 544-548.

[16] Mazzoleni S, Magni G, Toderini D. Effect of vitamin D3 seasonal supplementation with 1500â€‰IU/day in north Italian children (DINOS study). *Ital J Pediatr* 2019 Jan 28,**45**(1), 18.

[17] Chatfield SM, Brand C, Ebeling PR, Russell DM. Vitamin D deficiency in general medical inpatients in summer and winter. *Intern Med J* 2007 Jun,**37**(6), 377-382.

[18] Gallagher JC, Sai AJ. Vitamin D insufficiency, deficiency, and bone health. *J Clin Endocrinol Metab* 2010 Jun,**95**(6), 2630-2633.

[19] Ding C, Parameswaran V, Udayan R, Burgess J, Jones G. Circulating levels of inflammatory markers predict change in bone mineral density and resorption in older adults: a longitudinal study. *J Clin Endocrinol Metab* 2008 May,**93**(5), 1952-1958.

[20] Thompson R, Mitrou G, Brown S, et al. Major new review of global evidence on diet, nutrition and physical activity: A blueprint to reduce cancer risk. *Nutr Bull* 2018 Sep 1,**43**(3), 269-283.

[21] Shams-White MM, Brockton NT, Mitrou P, et al. Operationalizing the 2018 World Cancer Research Fund/American Institute for Cancer Research (WCRF/AICR) Cancer Prevention Recommendations: A Standardized Scoring System. *Nutrients* 2019 Jul 12,**11**(7).

[22] Epskamp S, Cramer A.O.J., Waldorp L.J., Schmittmann V.D., Borsboom D. qgraph: Network Visualizations of Relationships in Psychometric Data. *Journal of Statistical Software* 2012,**48**(4), 1-18.

[23] ter Braak CJF. Canonical Correspondence Analysis: A New Eigenvector Technique for Multivariate Direct Gradient Analysis. *Ecology* 1986 Oct 1,**67**(5), 1167-1179.

[24] Singh A, Shannon CP, Gautier B, et al. DIABLO: an integrative approach for identifying key molecular drivers from multi-omics assays. *Bioinformatics* 2019 Sep 1,**35**(17), 3055-3062.

[25] Rohart F, Gautier B, Singh A, Le Cao KA. mixOmics: An R package for 'omics feature selection and multiple data integration. *PLoS Comput Biol* 2017 Nov,**13**(11), e1005752.

[26] Vanderweele TJ, Vansteelandt S. Odds ratios for mediation analysis for a dichotomous outcome. *Am J Epidemiol* 2010 Dec 15,**172**(12), 1339-1348.

[27] Valeri L, Vanderweele TJ. Mediation analysis allowing for exposure-mediator interactions and causal interpretation: theoretical assumptions and implementation with SAS and SPSS macros. *Psychol Methods* 2013 Jun,**18**(2), 137-150.

[28] Liu D, Jiang XY, Zhou LS, Song JH, Zhang X. Effects of Probiotics on Intestinal Mucosa Barrier in Patients With Colorectal Cancer after Operation: Meta-Analysis of Randomized Controlled Trials. *Medicine (Baltimore)* 2016 Apr,**95**(15), e3342.

[29] Zou S, Fang L, Lee MH. Dysbiosis of gut microbiota in promoting the development of colorectal cancer. *Gastroenterol Rep (Oxf)* 2018 Feb,**6**(1), 1-12.

[30] Ley RE, Turnbaugh PJ, Klein S, Gordon JI. Microbial ecology: human gut microbes associated with obesity. *Nature* 2006 Dec 21,**444**(7122), 1022-1023.
